# Chronic lateral ankle instability using anterior tibiofibular ligament distal fascicle transfer augmentation repair: an anatomical, biomechanical, and histological study

**DOI:** 10.3389/fbioe.2024.1326036

**Published:** 2024-03-07

**Authors:** Ruihan Wang, Yingqiu Yang, Guixuan You, Lei Huang, Xin Zhou, Songtao Jiang, Houyin Shi, Guoyou Wang, Lei Zhang

**Affiliations:** ^1^ School of Physical Education, Southwest Medical University, Luzhou, China; ^2^ Department of Rehabilitation, Yibin Integrated Traditional Chinese and Western Medicine Hospital, Yibin, China; ^3^ Department of Orthopedics, The Affiliated Traditional Chinese Medicine Hospital, Southwest Medical University, Luzhou, China; ^4^ Center for Orthopedic Diseases Research, The Affiliated Traditional Chinese Medicine Hospital, Southwest Medical University, Luzhou, China; ^5^ Luzhou Key Laboratory of Orthopedic Disorders, Luzhou, China; ^6^ School of Clinical Medicine, Southwest Medical University, Luzhou, China

**Keywords:** anterior tibiofibular ligament distal fascicle transfer, anterior talofibular ligament, anterior tibiofibular ligament distal fascicle, anatomy, biomechanics, histology

## Abstract

**Background:** The transfer of the anterior tibiofibular ligament distal fascicle (ATiFL-DF) for the augmentation repair of the anterior talofibular ligament (ATFL) shows potential as a surgical technique. However, evidences on the benefits and disadvantages of this method in relation to ankle joint function are lacking.

**Purpose:** This study aimed to provide comprehensive experimental data to validate the feasibility of ATiFL-DF transfer augmentation repair of the ATFL.

**Methods:** This study included 50 embalmed ankle specimens to measure various morphological features, such as length, width, thickness, and angle, for evaluating similarities between the ATiFL-DF and ATFL. Furthermore, 24 fresh-frozen ankle specimens were examined for biomechanical testing of the ATiFL-DF transfer augmented repair of the ATFL. Finally, 12 pairs of ATiFL-DF and ATFL tissues from fresh-frozen ankle specimens were treated with gold chloride staining to analyze mechanoreceptor densities.

**Results:** Anatomical studies found that the lengths and thicknesses of the ATFL and ATiFL-DF are similar. Biomechanical outcomes showed that performing ATiFL-DF transfer for ATFL repair can improve the stability of the talus and ankle joints. This is evident from the results of the anterior drawer, axial load, and ultimate failure load tests. However, performing ATiFL-DF transfer may compromise the stability of the distal tibiofibular joint, based on the Cotton and axial load tests at an external rotation of 5°. Analysis of the histological findings revealed that mechanoreceptor densities for four types of mechanoreceptors were comparable between the ATiFL-DF and ATFL groups.

**Conclusion:** ATiFL-DF transfer is a viable method for augmenting ATFL repair. This technique helps to improve the stability of the talus and ankle joints while compensating for proprioception loss. Although ATiFL-DF transfer augmented repair of the ATFL may negatively affect the stability of the distal tibiofibular joint, this procedure can enhance the stability of the talus and ankle joints.

## 1 Introduction

The anterior talofibular ligament (ATFL), the posterior talofibular ligament, and the calcaneofibular ligament are important ligaments that provide stability to the lateral ankle joint (Clanton et al., 2014). Ankle sprains are the most common cause of ATFL injuries. If not treated promptly and properly after injury, repeated ATFL damage may result in chronic lateral ankle instability (CLAI) (Pacheco et al., 2021). Therefore, appropriate treatment should be performed promptly to reduce the long-term complications of ATFL injuries. Surgery may be required for ligament repair or reconstruction in patients with ankle instability (Tourné and Mabit, 2017). Both open and arthroscopic surgical techniques are effective for treating CLAI (Guelfi et al., 2018), although arthroscopy offers some advantages in terms of managing postoperative complications. Currently, the standard surgical option for CLAI is arthroscopic Broström-Gould repair (Woo et al., 2020); however, long-term follow-up shows that patients with CLAI undergoing the Brostrom-Gould procedure may experience recurrence (Petrera et al., 2014). The poor outcomes may be explained by the fact that the natural stiffness of the ligament can only be restored by up to 50% with this technique (Waldrop et al., 2012).

Comprehensive studies on ATFL repair have identified some limitations of surgical reconstruction and repair (Feng et al., 2020). Cabuk et al. (Çabuk and Kuşku Çabuk, 2016), reported that the cruciate ligaments and popliteus tendon in the knee have a higher density of mechanoreceptors than tendons, such as the gracilis. This implies that tendons and ligaments serve distinct biological functions and that maintaining ligaments is crucial for proper joint proprioception. Recently, researchers proposed using the distal fascicle of the anterior tibiofibular ligament (ATiFL) to augment the ATFL and increase postoperative ankle joint stability (Vega et al., 2020; Tian et al., 2022; Xiao et al., 2022). The ATiFL distal fascicle (ATiFL-DF) is situated at the tibiofibular syndesmosis, also called the Bassett ligament (Fisher et al., 2021). The ligament is inside the joint capsule and shares potential anatomical and biomechanical similarities with the ATFL (Vega et al., 2020). Its removal does not seem to have a considerable impact on joint stability (Akseki et al., 1999). In previous studies on ATiFL-DF transfer augmented repair, the focus was primarily on the efficacy of treatment and on ultimate failure load and biomechanics (Vega et al., 2020; Tian et al., 2022; Xiao et al., 2022).

This study aimed to (1) analyze the similarity of the anatomical morphology (angle, length, width, and thickness) of the ATFL and ATiFL-DF, (2) analyze the biomechanical differences (anterior drawer, Cotton, axial load at different positions, and ultimate failure load tests) between arthroscopic ATiFL-DF transfer for augmented repair of the ATFL and ATFL repair, and (3) compare the density of mechanoreceptors in the ATFL and ATiFL-DF. We performed anatomical measurements of embalmed ankle specimens, biomechanical experiments on fresh-frozen ankle specimens, and modified gold chloride staining of the ligament tissue. We aimed to provide comprehensive experimental data to validate the feasibility of enhancing ATFL repair using ATiFL-DF transfer.

## 2 Methods

This study complied with protocols approved by the Medical Ethics Review Board of The Affiliated Traditional Chinese Medicine Hospital, Southwest Medical University (KY2022041).

### 2.1 Specimen

Fifty adult embalmed ankle specimens and 24 adult fresh-frozen ankle specimens were obtained from the Department of Anatomy, Southwest Medical University. Computed tomography was performed on all specimens to ensure eligibility. The exclusion criteria were as follows: (1) ankle ligament injury, (2) congenital malformation, (3) ankle gout, and (4) ankle fractures.

### 2.2 Anatomic measurement

**FIGURE 1:**
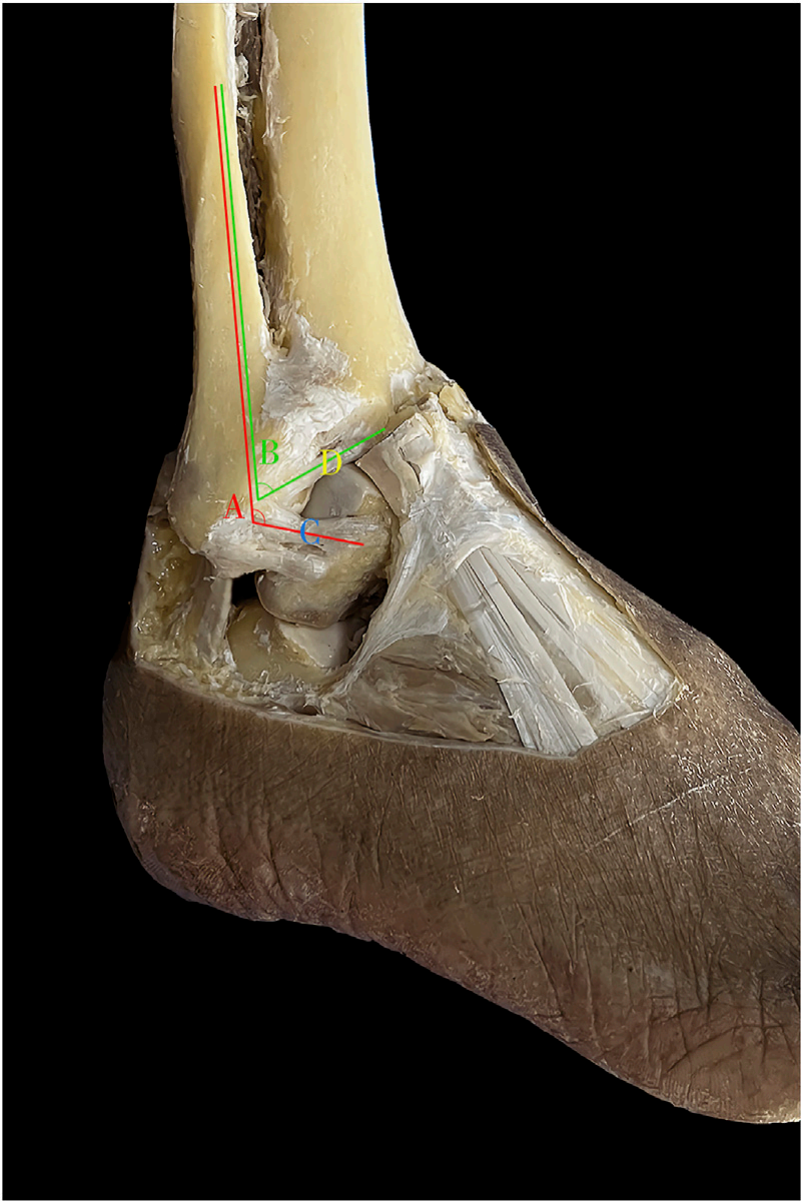
Anatomic measurement. A: angle between the anterior talofibular ligament and fibular axis; B: angle between the anterior tibiofibular ligament’s distal fascicle and fibular axis; C: length, width, and thickness of anterior talofibular ligament; D: length, width, and thickness of anterior tibiofibular ligament distal fascicle.

A total of 50 embalmed ankle specimens were used. An incision was made with a scalpel from 15 cm above the distal tibia and the fibular head. Sagittal incisions were made from the anterior and posterior edges of the incision, extending to the dorsal metatarsophalangeal joint and the lateral edge of the foot, respectively, to expose the anterolateral aspect of the foot entirely. Length was measured in millimeters (mm) and angles were measured in degrees (°). Considering that the ATFL has different fascicles, only the superior fascicle was measured when two or three fascicles were present. Various ATFL and ATiFL-DF parameters were measured (Figure 1). The parameter averages were obtained from two investigators.

### 2.3 Biomechanical experiment

#### 2.3.1 Model establishment

**FIGURE 2:**
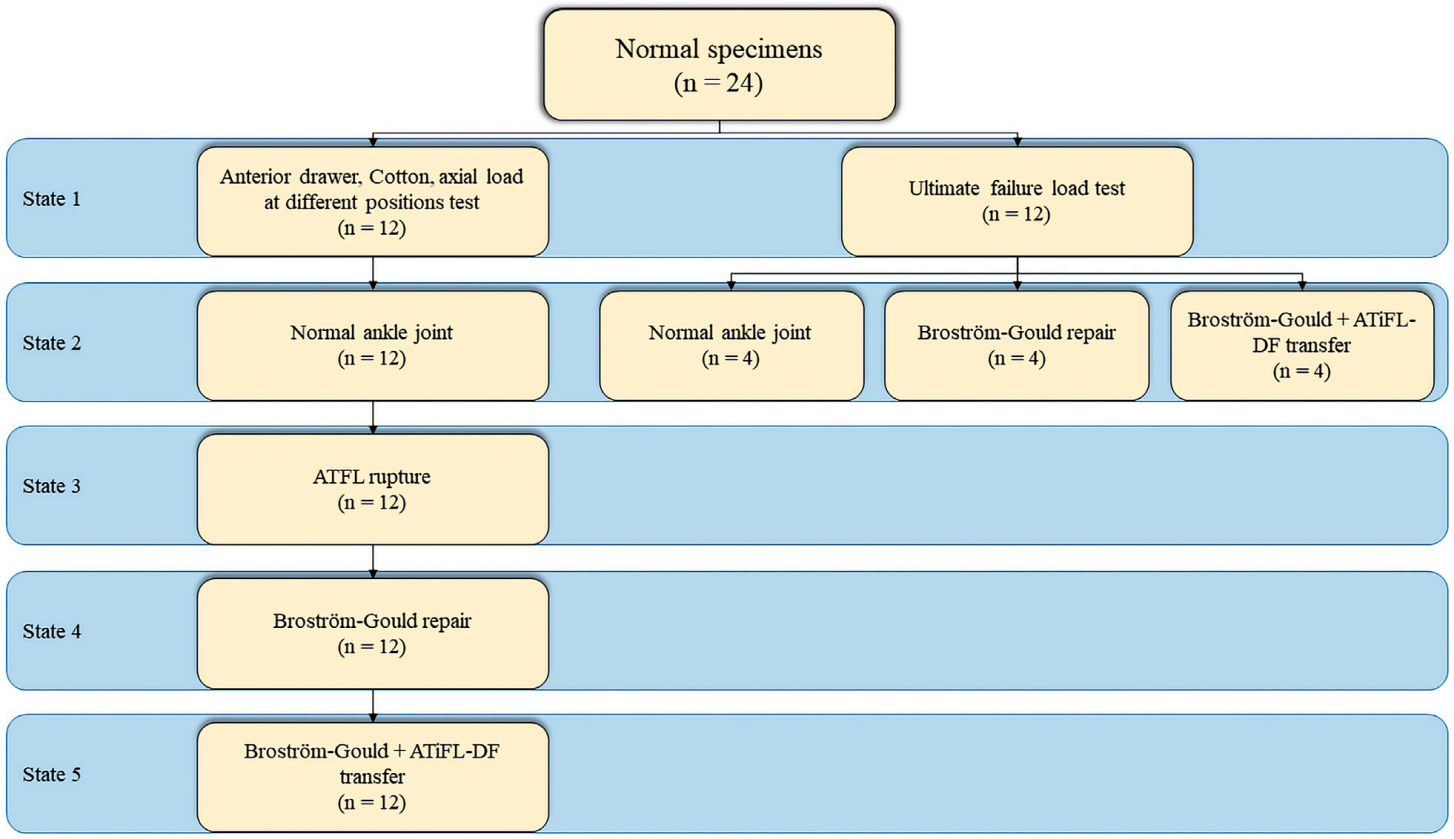
Steps of biomechanical testing.

Two experiments involving a total of 24 fresh-frozen ankle joint specimens (12 in each experiment) were conducted. In the first experiment, 12 specimens were created in different states: Group A, normal ankle joint; Group B, ATFL rupture; Group C, Broström-Gould repair; and Group D, Broström-Gould+ATiFL-DF transfer (Figure 2). In the second experiment, 12 specimens were divided into three groups (normal ankle joint, Broström-Gould repair, and Broström-Gould+ATiFL-DF transfer), with four specimens in each group used for the ultimate failure load test (Figure 2).

**FIGURE 3:**
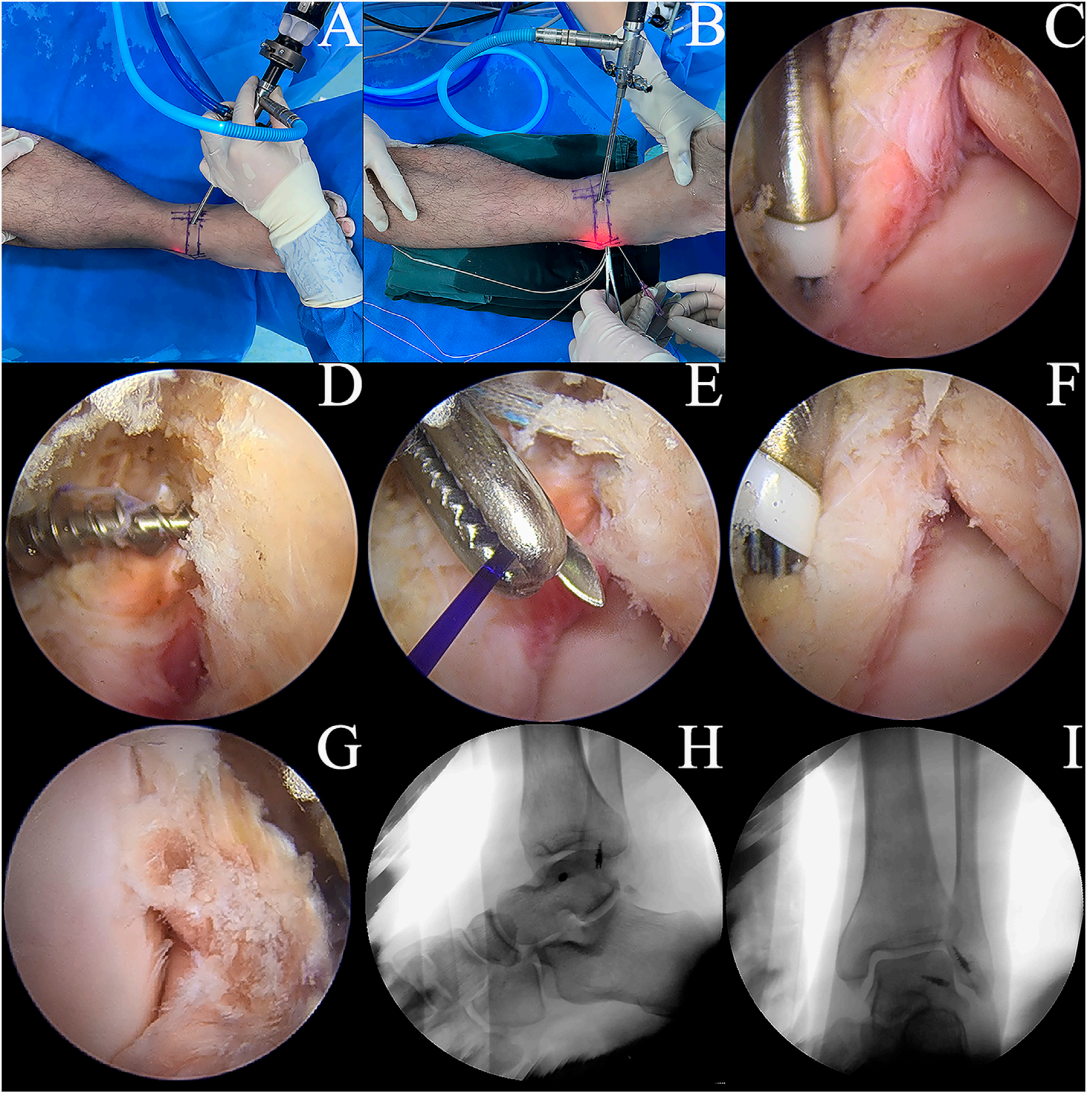
Model development. **(A,B)** ankle arthroscopic manipulation. **(C)** A plasma electric knife was used to cut the ATFL at the fibula attachment end of the ATFL. **(D)** implantation of a metal suture anchor at the attachment end of the talus. **(E)** suturing of the ATFL and the inferior extensor retinaculum. **(F)** application of the Broström-Gould procedure to the repaired ATFL. **(G)** the ATiFL-DF was cut at the attachment end of the tibia using a plasma electric knife and immediately rotated downward and fixed to the talar neck by transposition of the metal suture anchor. **(H,I)** postoperative lateral and orthopantomogram of the ankle.

Group B: The ankle specimen was secured to the operating table, and specific areas of the skin surface, such as the security zone, ATFL, and fibula, were marked. Two types of access, median and anterolateral, were established using a non-traction technique focused on ankle plantar flexion. The internal structure of the ankle was viewed directly by arthroscopy (Figures 3A, B). To prepare the ATFL rupture model, the ATFL was separated from the fibular insertion point using a plasma knife via an anterolateral proximal portal (Figure 3C).

Group C: Based on the Group B model, a suture anchor (HealFix^®^, Rejoin, Hangzhou, China) was inserted into the fibular attachment through the anterolateral proximal portal. The suture was passed through the distal outer part of the ATFL to the proximal inner part, with arthroscopic visualization of the ligament. Then, the inferior extensor retinaculum was pierced using the same suture and fixed (Figures 3D–F).

Group D: Based on the Group C model, the ATiFL-DF was transferred to the talar neck under arthroscopy (Figure 3G). The tibial attachment end of the ATiFL-DF was located using a probing hook. The excess synovial tissue around the ligament was cleared using a planer knife to expose the ATiFL-DF fully. The ATiFL-DF was carefully cut at the tibial attachment end using a plasma electric knife and was removed with the small bone fragment of the tibia for maintaining its integrity. Once the ATiFL-DF was separated from the attachment end of the tibia, it was immediately turned downward. The area at the talar neck was cleared with a planer to allow placement of the metal anchor. Caution was taken to prevent damage to the cartilage, ensuring that the anchor placement site was positioned precisely at the exposed triangular area on the front and lateral aspects of the talus. The anchor was inserted into the tunnel with a bone hammer, and a positioning needle threaded with a polydioxanone (PDS) suture was inserted through the ATiFL-DF break end from the anterolateral portal. Next, a straight clamp was inserted from the anterolateral portal to grasp the PDS suture and pull it out of the body. The anchor staple suture was passed through the loop of the PDS suture and pulled back. We ensured that the ankle was in a 90° position or slight plantarflexion without inversion or valgus during surgery. Finally, the same color suture was knotted outside the body, and the excess suture was cut off using a knot pusher to push the knot to the anchor nail implantation. A C-arm-shaped radiograph was used to capture lateral and frontal radiographs of the ankle to verify the success of the procedure (Figures 3H, I). All procedures were performed by the same experienced orthopedic surgeon (Lei Zhang).

#### 2.3.2 Biomechanical testing

**FIGURE 4:**
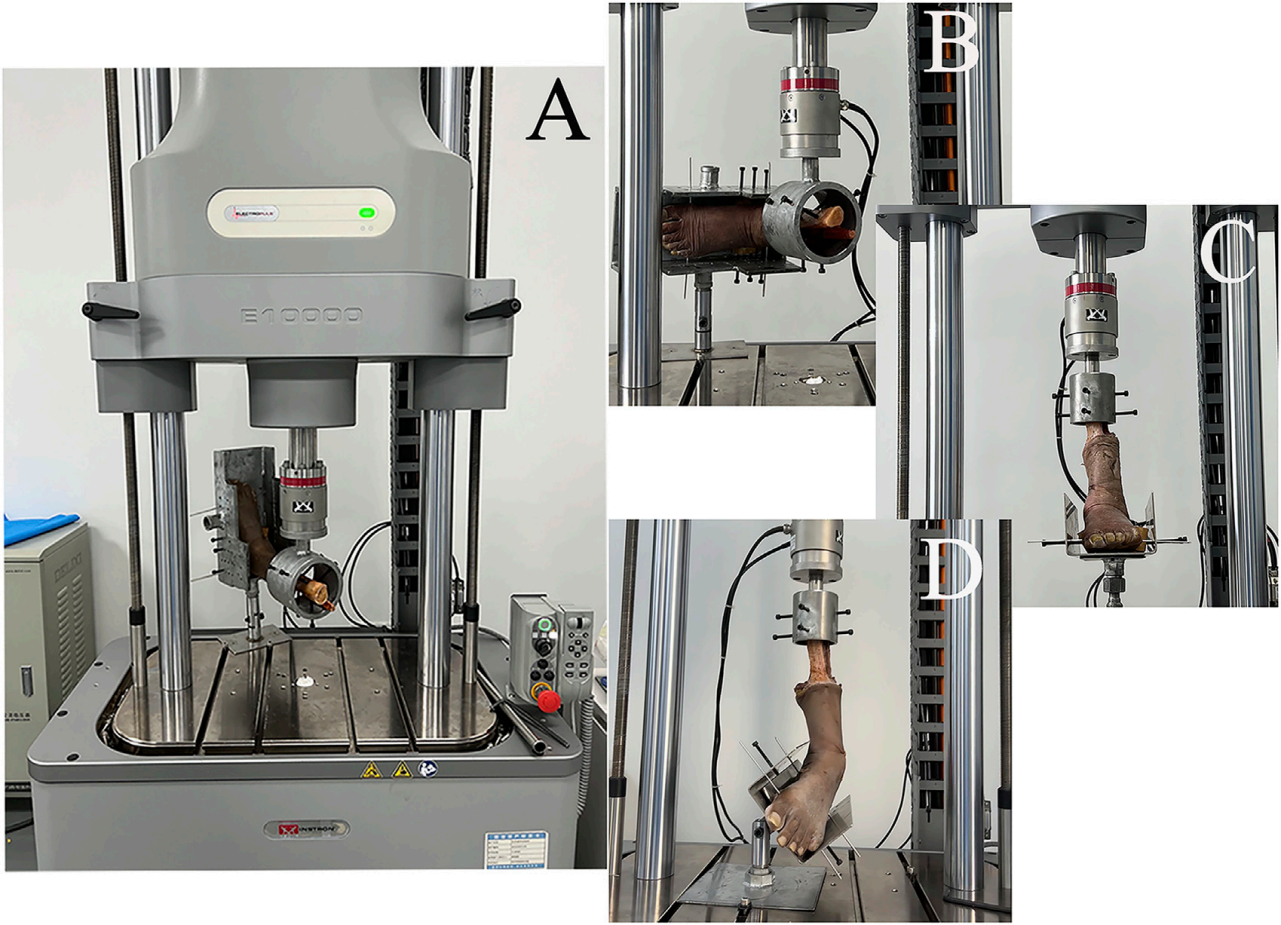
Biomechanical testing. **(A)** anterior drawer test; **(B)** Cotton test; **(C)** axial load test at different positions (plantarflexion 30°, dorsiflexion 20°, inversion 15°, eversion 10°, internal rotation 5°, and external rotation 5°); **(D)** ultimate failure loads.

All-Electric Dynamic and Fatigue Test Systems (ElectroPuls E10000, Instron, Boston, Massachusetts, United States) were used for the biomechanical experiments. The specimens were placed in a biomechanical testing machine. The foot of each specimen was firmly fixed to the clamp device and locked into the fixation bracket to prevent loosening during the experiment. The proximal end of the tibia was held firmly using a fixation device. All loads were applied at 10 N/s. Biomechanical testing included an anterior drawer test of the ankle at neutral and 30° of plantar flexion, a Cotton test at neutral, an axial pressure test at different positions, and an ultimate failure load test (Figure 4). We simulated the anterior drawer test by applying a 100 N anterior-posterior load on the tibia with the foot fixed (Figure 4A). The Cotton test was simulated by applying a 100 N medial-lateral load on the tibia with the foot fixed, resulting in separation between the tibia and fibula (Figure 4B). A 500 N axial load was applied with 30° of static plantar flexion, 20° of dorsiflexion, 15° of inversion, 10° of eversion, 5° of internal rotation, and 5° of external rotation (Figure 4C). To simulate the ultimate failure loads of an ankle sprain, the specimen was initially positioned at 10° of plantar flexion and 30° of inversion, with the inversion axis set to a flexible state. The axial load was applied continuously to the tibia and fibula until the lateral ankle ligament ruptured (Figure 4D). All data were collected and recorded using All-Electric Dynamic and Fatigue Test Systems, with the final output recorded in an Excel spreadsheet.

#### 2.3.3 Histological observation

**FIGURE 5:**
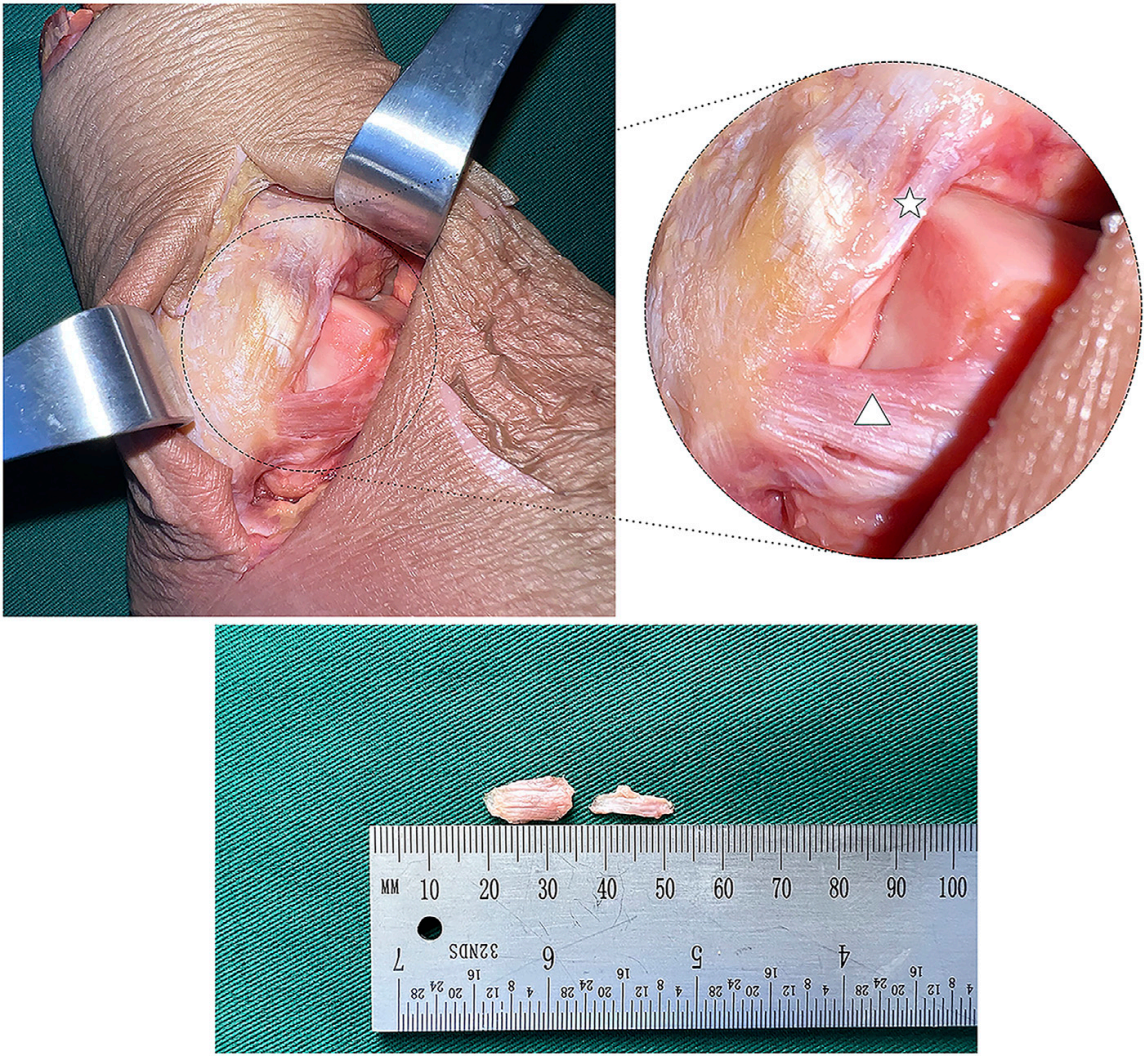
Anatomical picture of the lateral ankle joint (white star: anterior tibiofibular ligament’s distal fascicle; white triangle: anterior talofibular ligament).

After the biomechanical test, the ATiFL-DF and ATFL (average thickness, 2.6 mm) were dissected from 12 fresh-frozen ankle specimens (Figure 5). The tissues were processed and stained using a modified gold chloride technique (Zimny et al., 1985). (1) The dissected ligaments were placed in 0.9% sodium chloride and stored in a refrigerator. (2) The specimens were removed and placed in a mixture of fresh lemon juice and 88% formic acid (Macklin, China) for three 15-min cycles in a dark room. (3) The specimens were removed, and the surface liquid was dried with filter paper before being placed in a 1% gold chloride solution (Huaxia, China) for 1.5 h in a dark room. (4) The specimens were removed, and the surface liquid was dried with filter paper before being placed in 25% formic acid and left in a dark room for 15–16 h (5) The specimens were placed in a mixture of 30% sucrose (Leagene, China) and optimal cutting temperature compound (OCT) (Sakura, United States) for 2 h (6) The tissue was embedded using OCT, frozen at −80°C, sliced into 30 μm-thick sections, and air-dried for 1 day. (7) The samples were dehydrated in 60%, 70%, 90%, and 100% ethanol (Cologne, China) for 3 min each. Finally, the proximal, middle, and distal portions of the ligament slices were marked. All sections were viewed using an optical microscope (DM500; Leica Microsystems, Germany) and observations were recorded.

All slices were classified and counted using the mechanoreceptor classification system described by Freeman and Wyke (Freeman and Wyke, 1966). Because we could not fully observe all four types of mechanoreceptors from the proximal, middle, and distal portions of the ligament, we planned to locate the densest areas of each mechanoreceptor type in three consecutive slices and calculate their densities. We calculated the density of the mechanoreceptors per 1 mm^2^ by dividing the total number of receptors per 1 mm^2^ by the square of the average thickness of the specimen sections (Yeo et al., 2016).

### 2.4 Statistical analysis

Normality tests were performed using the Shapiro–Wilk test. The Levene test was employed to analyze the homogeneity of variance. If the parametric test hypothesis was satisfied, two independent sample t-tests were used. However, when the hypotheses did not meet the criteria of the parametric test, the Mann–Whitney U test was preferred. Two independent sample t-tests were used for ligament morphological measurement analysis. The Mann-Whitney U test was chosen for mechanoreceptor density analysis because the sample size was less than 30.

To match the repeated measures experimental design (Stake et al., 2023), random-intercept linear mixed-effects models were used to compare the displacement during the anterior drawer test at neutral and at 30° of plantar flexion, the Cotton test, and the axial load test at different positions (30° plantar flexion, 20° dorsiflexion, 15° inversion, 10° eversion, 5° internal rotation, and 5° external rotation). The restricted maximum likelihood method was performed to estimate the model. The Bonferroni–Dunn method was performed to make pairwise comparisons among states with reported marginal means. The residual diagnostics were examined to guarantee the adequacy of the model and fulfill the assumptions. Statistical significance was set at a *p*-value of <.05. For the ultimate failure load test, the Bonferroni–Dunn method of ANOVA was used for pairwise comparison. SPSS (Version 24, IBM, United States) and graphics software OriginPro (Version 2021, OriginLab, United States) were used for all statistical analyses and plots.

## 3 Results

### 3.1 Morphological measurements

The anatomical parameters of the ATFL and ATiFL-DF ligaments were analyzed in 50 embalmed ankle specimens with intact ligaments. The study found that the angle and width measurements showed significant differences (*p* < .05). However, there were no significant differences (*p* > .05) in length and thickness measurements, indicating that they were similar (Table 1).

**TABLE 1:** Measurement of anatomical parameters.

Angle (°)		Length (mm)		Width (mm)		Thickness (mm)	
A	105.80 ± 4.71	C1	16.35 ± 1.45	C2	6.71 ± 1.95	C3	1.32 ± 0.36
B	53.57 ± 8.00	D1	15.81 ± 1.57	D2	4.27 ± 1.20	D3	1.27 ± 0.31
*p*	0.000	*p*	0.073*	*p*	0.000	*p*	0.504*

*p > 0.05 implied anatomical similarity; Data described as mean ± SD (standard deviation).

### 3.2 Anterior drawer test

**FIGURE 6:**
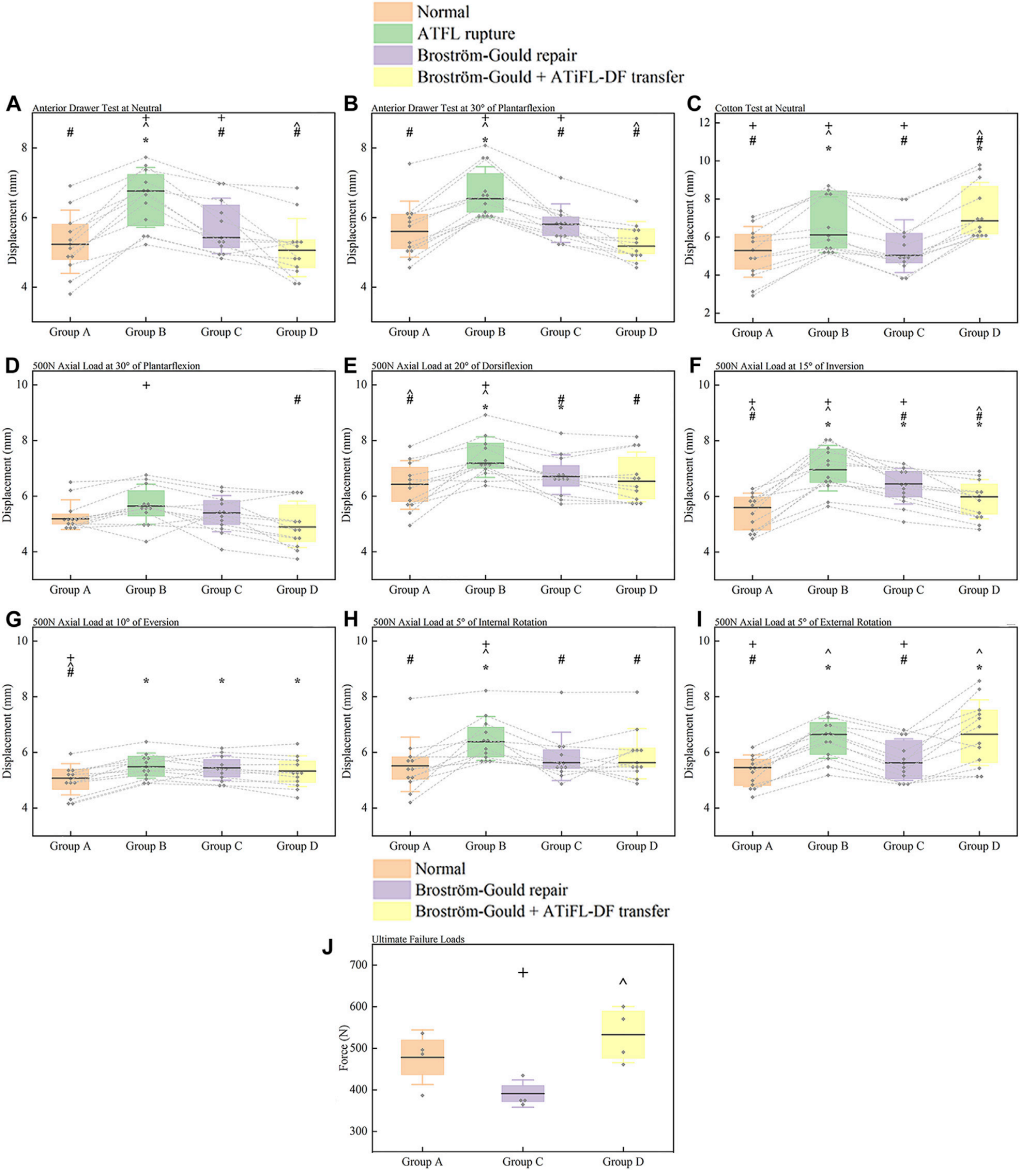
Box plots. **(A)** anterior drawer test at neutral; **(B)** anterior drawer test at 30° of plantarflexion; **(C)** Cotton test at neutral; **(D)** axial load test at 30° of plantarflexion; **(E)** axial load test at 20° of dorsiflexion; **(F)** axial load test at 15° of inversion; **(G)** axial load test at 10° of eversion; **(H)** axial load test at 5° of internal rotation; **(I)** axial load test at 5° of external rotation; **(J)** ultimate failure loads test of an ankle sprain. For anterior drawer test at neutral, 30° plantarflexion, Cotton test at neutral, and axial load test of 500 N at different positions, all measured values were displacement distances, and the results were represented as median (horizontal lines), box (interquartile distance of 25th and 75th percentiles), standard deviation (whisker lines), and individual specimen observations (diamond-shaped point). Each dotted line was the same specimen. ^*^ *p* < .05 vs. normal state (Group A). ^#^ *p* < .05 vs. ATFL rupture (Group B). ^^^ *p* < .05 vs. Broström-Gould repair (Group C). ^+^ *p* < .05 vs. Broström-Gould+ATiFL-DF transfer (Group D). For ultimate failure loads test, all measured values were force (N), and the results were represented as mean (horizontal lines), box (interquartile distance of 25th and 75th percentiles), standard deviation (whisker lines), and individual specimen observations (diamond-shaped point). ^^^ *p* < .05 vs. Broström-Gould repair (Group C). ^+^ *p* < .05 vs. Broström-Gould+ATiFL-DF transfer (Group D).

The anterior drawer test was performed at neutral and plantarflexion at 30°. The ATFL rupture state showed greater displacement in comparison to the other state (*p* < .05). Displacement of Broström-Gould + ATiFL-DF transfer was less than that observed with the Broström-Gould repair (*p* < .05) (Figures 6A, B).

### 3.3 Cotton test

Compared with the normal state, ATFL rupture and Broström-Gould + ATiFL-DF transfer showed considerably greater displacement during the Cotton test (*p* < .05). However, the Broström-Gould + ATiFL-DF transfer showed more significant displacement than that observed with any other state (*p* < .05) (Figure 6C).

### 3.4 Axial load test at different positions

At an axial load of 500 N at 30° plantarflexion, compared with the ATFL rupture state, the Broström-Gould + ATiFL-DF transfer demonstrated less displacement (*p* < .05). However, no significant differences were found between the normal state, ATFL rupture, and Broström-Gould repair (Figure 6D).

At an axial load of 500 N at 20° dorsiflexion, compared with the normal state, ATFL rupture and Broström-Gould repair showed considerably more displacement (*p* < .05). However, no significant difference was found between the normal state and Broström-Gould + ATiFL-DF transfer (Figure 6E).

At an axial load of 500 N at 15° inversion, compared with the normal state, other states showed more significant displacement (*p* < .05). Compared to the ATFL rupture and Broström-Gould repair, the Broström-Gould + ATiFL-DF transfer demonstrated the least displacement (*p* < .05) (Figure 6F).

At an axial load of 500 N at 10° eversion, compared with the normal state, other states showed more significant displacement (*p* < .05). However, no significant difference was found between ATFL rupture, Broström-Gould repair, and Broström-Gould + ATiFL-DF transfer (Figure 6G).

At an axial load of 500 N at 5° internal rotation, compared with the other state, ATFL rupture showed considerably more displacement (*p* < .05). However, no significant difference was found between ATFL rupture, Broström-Gould repair, and Broström-Gould + ATiFL-DF transfer (Figure 6H).

At an axial load of 500 N at 5° external rotation, compared with the normal state, ATFL rupture and Broström-Gould + ATiFL-DF transfer showed considerably greater displacement (*p* < .05). The normal state and Broström-Gould repair showed smaller displacement than ATFL rupture (*p* < .05); however, no significant difference was found between ATFL rupture and Broström-Gould + ATiFL-DF transfer (Figure 6I).

### 3.5 Ultimate failure load test

To simulate the ultimate failure loads of the ankle sprain, no significant difference was found between the Broström-Gould repair and Broström-Gould + ATiFL-DF transfer groups and the normal group. However, the Broström-Gould + ATiFL-DF transfer showed more significant ultimate failure load than Broström-Gould repair (*p* < .05) (Figure 6J).

### 3.6 Mechanoreceptor density

**FIGURE 7:**
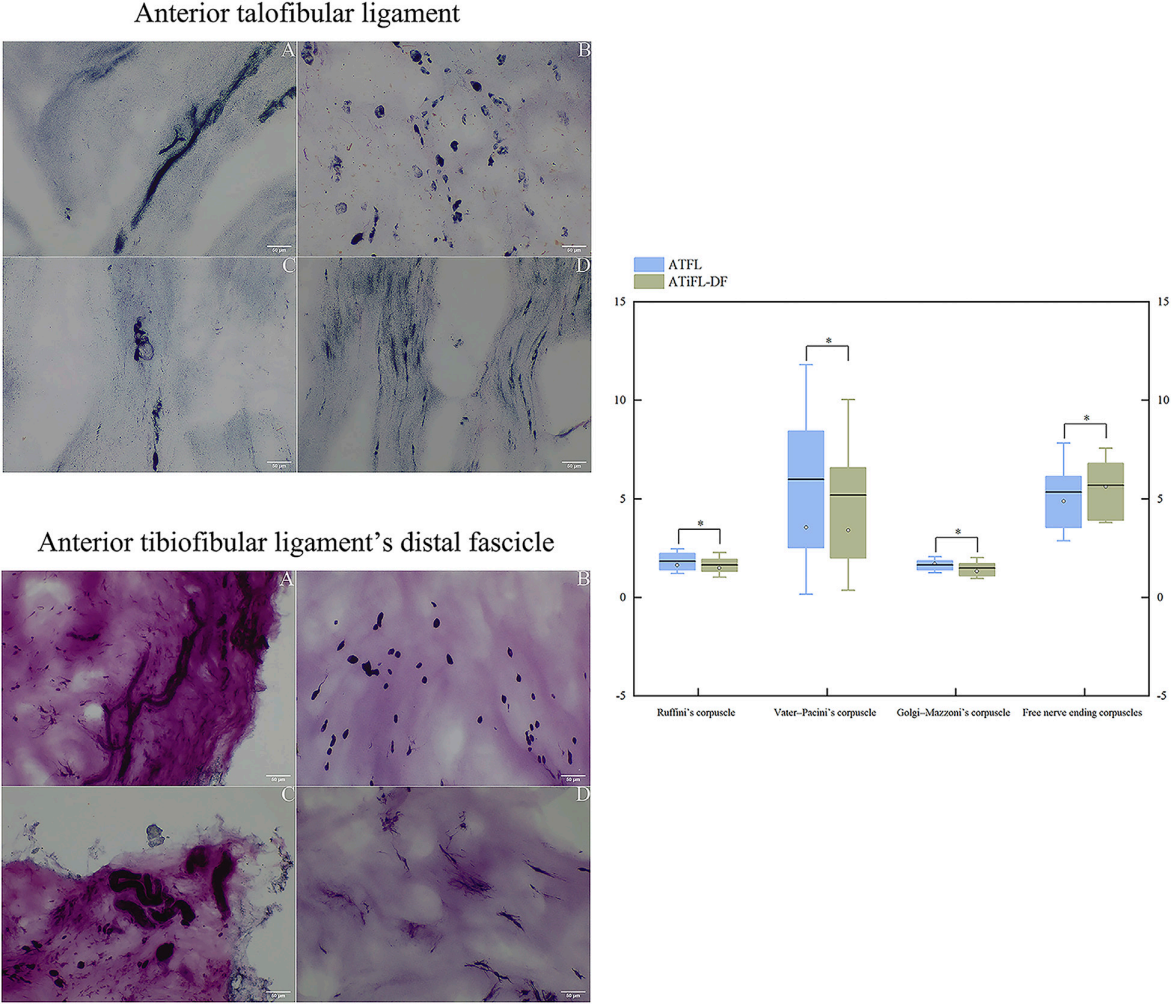
Photomicrographic images (200×) of the anterior talofibular ligament and anterior tibiofibular ligament’s distal fascicle using modified gold chloride staining. A: Ruffini’s corpuscle; B: Vater–Pacini’s corpuscle; C: Golgi-Mazzoni’s corpuscle; D: free nerve ending corpuscles. Box plots of comparison of the densities of mechanoreceptors between ATFL and ATiFL-DF are shown. The results are represented as mean value (horizontal lines), standard deviation (whisker lines), median (dot), and box (interquartile distance of 25th and 75th percentiles). ^*^ *p* > .05 Mechanoreceptor density was similar between ATFL and ATiFL-DF.

Based on Freeman and Wyke’s classification of mechanoreceptors, four mechanoreceptor types were observed in ATFL and ATiFL-DF tissues. Type I corpuscles of Ruffini appeared as scattered groupings with branches, whereas clustering occurred in Type II Vater-Pacini corpuscles that displayed an ovoid or cylindrical morphology and were enclosed. The features of Type III Golgi-Mazzoni corpuscles were indistinct, with a helical, fusiform, or spindle-like shape. Finally, Type IV corpuscles had no clear structure and consisted of thin and lengthy free nerve endings (Figure 7). Compared with Ruffini and Golgi-Mazzoni’s corpuscles, the Vater-Pacini and free nerve ending corpuscles demonstrated a slightly higher density. Additionally, when analyzing the four types of mechanoreceptors, no significant differences (*p* > .05) were observed between the ATFL and ATiFL-DF (Figure 7).

## 4 Discussion

The most important finding of this study is that the ATiFL-DF transfer is a viable method for augmenting anterior talofibular ligament repair based on anatomical, biomechanical, and histopathological data.

Anatomically, it was evident that the ATiFL-DF and superior fascicle of the ATFL are located in proximity. In addition, these two ligaments exhibit numerous similarities, including joint position, origin on the fibula, and length and width measurements. Due to the unpredictable bifurcation of the ATFL, we did not differentiate between its various fascicles during measurement. Instead, we measured the superior fascicle when two or three were present. Thus, while measuring the ligaments in the 50 embalmed ankle specimens, we noted that the lengths and thicknesses of the ATFL and ATiFL-DF were similar. Bassett et al. (Bassett et al., 1990) found that 71% of the patients with lateral ankle instability had a worn cartilage area on the lateral of the talus. This suggested that lateral instability resulting from injury to the ATFL could lead to pathological ATiFL-DF, causing ankle pain. Akseki et al. (Akseki et al., 1999) performed arthroscopic ATiFL-DF removal in 21 patients with ankle impingement after ankle sprains. In all cases, an impingement ATiFL-DF was identified and its removal did not affect ankle stability. Rasmussen et al. (Rasmussen et al., 1982) found that after iatrogenic transection of the ATiFL-DF, there was only a slight increase in ankle lateral rotation by an average of 1.5°, indicating that it contributed minimally to ankle stability.

In the surgical treatment of chronic ankle instability, the Broström technique for ATFL repair has been widely accepted as the “gold” standard (Guillo et al., 2013). This procedure is commonly performed using arthroscopic examination and Broström-Gould repair.(Han et al., 2023). This anatomical repair with a direct overlapping suture and further suturing under the lateral extensor muscle reinforces ankle repair (Aydogan et al., 2006). Improved repair of the inferior extensor retinaculum provides initial protection and improved repair of the ATFL for the rotational function of the joint (Dalmau-Pastor et al., 2018). In addition, inferior extensor retinaculum augmentation may be performed using the total inner gloving technique to avoid nerve injury (Yang et al., 2022). In 2019, Vega et al. (Vega et al., 2020) performed total arthroscopic ATiFL-DF transfer enhancement using five fresh-frozen ankle joint specimens and reported that transfer via the anterior-lateral portal did not harm the superficial peroneal nerve. This technique is a promising surgical method, as ATiFL-DF transfer avoids the need for tendon harvesting or allografts and can further enhance ATFL repair.

We conducted biomechanical assessments by placing the foot in various positions to replicate routine clinical tests such as the anterior drawer, Cotton, and axial load tests. Moreover, ankle sprains were simulated, and the ultimate failure loads were recorded. In the anterior drawer test, the average displacement of the Broström-Gould + ATiFL-DF transfer in the neutral or dorsiflexion position was slightly lower than that in the normal state. During the axial load test, the average displacement of the Broström-Gould + ATiFL-DF transfer was less than that of the Broström-Gould repair. The displacement at 30° plantar flexion was smaller than that in the normal group, whereas for the other positions, the displacement was greater than that in the normal group. Furthermore, the ultimate failure load of Broström-Gould + ATiFL-DF transfer can also withstand an average of 54.39 N more than the normal state. In a cohort study that compared ATiFL-DF transfer and InternalBrace^TM^ ligament reconstruction, Tian et al. (Tian et al., 2022) discovered that 1 year after recovery, ATiFL-DF transfer could yield positive clinical outcomes, and patients who underwent this technique exhibited significant improvement in AOFAS scores compared with the control group. This is consistent with our experimental biomechanical findings, demonstrating that this method is viable for surgeons and patients. However, it should be noted that the Cotton test revealed that the average displacement of the ATiFL-DF increased by 2.16 mm after transposition compared with the normal state (Group A) (Table 2). Simultaneously, we observed that the axial load during 5° external rotation resulted in an average increase in the 1.36-mm displacement of ATFL repair + ATiFL-DF transfer compared with that in the normal state. This finding implies that ATiFL-DF transfer could affect the stability of the distal tibiofibular joint. We acknowledge that our biomechanical findings did not consider the effects of biological healing. Although this does not fully demonstrate a sustained healing response after surgery, it underscores the significance of postoperative rehabilitation. In a case study, Coetzee et al. (Coetzee et al., 2018) observed that InternalBrace^TM^ augmentation surgery paired with an accelerated rehabilitation program yielded positive outcomes in preventing the recurrence of ankle joint instability in the short term. Past and present reports (Larkins et al., 2021) imply that stabilizing the talus by fixing the ankle joint in a neutral and dorsiflexed position after surgery aids protective rehabilitation and enhances clinical results.

**TABLE 2:** Ankle displacement (mm) and failure loads (N) with different tests and positions.

Test	Normal	ATFL rupture	Broström-gould repair	Broström-gould + ATiFL-DF transfer
Anterior Drawer at 0°	5.30 ± 0.91	6.58 ± 0.86	5.76 ± 0.80	5.14 ± 0.84
Anterior Drawer at 30°	5.66 ± 0.81	6.72 ± 0.73	5.83 ± 0.55	5.32 ± 0.56
Cotton Test at 0°	5.21 ± 1.33	6.64 ± 1.46	5.51 ± 1.39	7.37 ± 1.48
Axial Load at plantarflexion 30°	5.33 ± 0.53	5.71 ± 0.72	5.37 ± 0.65	4.99 ± 0.83
Axial Load at dorsiflexion 20°	6.40 ± 0.87	7.40 ± 0.73	6.77 ± 0.71	6.69 ± 0.89
Axial Load at inversion 15°	5.46 ± 0.65	7.01 ± 0.82	6.37 ± 0.64	5.90 ± 0.71
Axial Load at eversion 10°	5.03 ± 0.56	5.52 ± 0.45	5.44 ± 0.44	5.32 ± 0.55
Axial Load at internal rotation 5°	5.56 ± 0.98	6.50 ± 0.79	5.85 ± 0.87	5.95 ± 0.90
Axial Load at external rotation 5°	5.34 ± 0.57	6.50 ± 0.71	5.74 ± 0.74	6.70 ± 1.18
Ultimate Failure Loads	478.10 ± 65.63	-	390.76 ± 32.81	532.49 ± 67.68

ATFL, anterior talofibular ligament; ATFL-DF, anterior tibiofibular ligament’s distal fascicle; Data described as mean ± SD (standard deviation).

Our histological analysis identified four types of mechanoreceptors in the ATiFL-DF and ATFL. According to Freeman and Wyke’s classification (Freeman and Wyke, 1966), mechanoreceptors comprise the Ruffini, Vater-Pacini, and Golgi-Mazzoni corpuscles and free nerve endings. Type I Ruffini corpuscles respond to mechanical stress, have a low threshold and slow adaptation, and are categorized as static or dynamic. They play crucial roles in transmitting information related to joint movements; specifically, they provide information about the static position, changes in pressure within the joint, and the direction, amplitude, and velocity of joint movements. This information helps coordinate movements and maintain joint stability. Type II Vater-Pacini corpuscles have a low threshold and can rapidly adapt. They are activated when the joint movement starts or stops. Type III Golgi-Mazzoni corpuscles possess elevated thresholds and exhibit sluggish adaptation, responding when subjected to significant pressure or extreme motion. Type IV free nerve endings are sensory receptors that specifically detect noxious stimuli and exhibit high activation thresholds. These receptors are also referred to as non-adapting pain receptors (Freeman and Wyke, 1966; Cavalcante et al., 2004). In 2015, Yeo et al. (Yeo et al., 2016) conducted a histological study to determine the density of mechanoreceptors in the ATiFL-DF, ATFL, and synovium and found no significant difference in density among the three soft tissues. This finding is consistent with ours as we found no significant differences between the ATFL and ATiFL-DF. The primary role of the ATFL is preventing excessive inversion and plantarflexion of the ankle (Kobayashi et al., 2020). When the ATiFL-DF is transferred to the talus, its proprioceptive function is transferred to the talus, thereby increasing the stability of the ATFL to the ankle joint. Therefore, after an ATFL rupture, transferring the ATiFL-DF to the talus during Broström-Gould repair can further enhance the anterior and inversion stability of the ATFL.

The standard Broström-Gould procedure is used in patients with good quality of the ligament stump, minimal loss of proprioception, and low requirements in terms of physical activity. However, patients with chronic ATFL injury and impaired proprioception may benefit from ATiFL-DF transfer. Therefore, we recommend ATiFL-DF transfer in cases with ankle joint impingement and chronic ankle instability, instead of removing it. ATiFL-DF transfer can help resolve the ankle joint impact and maintain proprioception, thereby improving ankle joint stability. Furthermore, this procedure can avoid the creation of a periosteal flap, tendon, and other grafts, and other injuries. Compared with an internal brace, this procedure not only preserves proprioception to the greatest extent, but also avoids increased biomaterial implantation. Furthermore, enhanced repair using an internal brace with ATiFL-DF transfer may also be considered when repair of the ligament stump cannot be performed. However, the indications for this procedure require further exploration.

This study had several limitations. Using embalmed cadavers for the anatomical measurements of the ATFL and ATiFL-DF may have resulted in fixed ligaments that deviated from their actual state. Although cadaveric biomechanical studies can provide valuable information about the immediate strength of repair of ATFL injuries, they do not indicate the long-term healing response of ankle tissues. Hence, it is imperative to include a comprehensive postoperative rehabilitation program, irrespective of the type of surgery undertaken, for ATFL repair. Furthermore, performing sequential tests on these corpses could have caused the nearby soft tissues to stretch, gradually weakening them. The experimental procedures were performed with the tibia and foot rigidly secured to ensure consistency and prevent excessively tight repairs during laboratory trials. However, this method does not perfectly replicate real-world conditions. The identification of mechanoreceptors in measurements can be challenging due to the small size of free nerve endings, which makes it difficult to distinguish them from other nerve bundles. Furthermore, certain Ruffini corpuscles can resemble Golgi-Mazzoni corpuscles (Katonis et al., 1991).

## 5 Conclusion

The ATiFL-DF transfer is a viable method for augmenting ATFL repair. This biological enhancement technique helps improve the stability of the talus and ankle joints while compensating for the loss of proprioception. However, ATiFL-DF transfer may compromise the stability of the distal tibiofibular joint. A thorough clinical follow-up is required to assess long-term biological healing and kinematic and proprioceptive changes. Although ATiFL-DF transfer augmented repair of the ATFL may negatively affect the stability of the distal tibiofibular joint, this procedure can enhance the stability of the talus and ankle joints. These results indicate that patients with chronic ATFL injury and impaired proprioception may benefit from this procedure.

## Data Availability

The raw data supporting the conclusion of this article will be made available by the authors, without undue reservation.

## References

[B1] Akseki D. Pinar H. Bozkurt M. Yaldiz K. Araç S. (1999). The distal fascicle of the anterior inferior tibio-fibular ligament as a cause of anterolateral ankle impingement: results of arthroscopic resection. Acta Orthop. Scand. 70 (5), 478–482. 10.3109/17453679909000984 10622481

[B2] Aydogan U. Glisson R. R. Nunley J. A. (2006). Extensor retinaculum augmentation reinforces anterior talofibular ligament repair. Clin. Orthop. Relat. Res. 442, 210–215. 10.1097/01.blo.0000183737.43245.26 16394763

[B3] Bassett F. H. 3rd Gates H. S. 3rd Billys J. B. Morris H. B. Nikolaou P. K. (1990). Talar impingement by the anteroinferior tibiofibular ligament. A cause of chronic pain in the ankle after inversion sprain. J. bone Jt. Surg. Am. volume 72 (1), 55–59. 10.2106/00004623-199072010-00009 2295673

[B4] Çabuk H. Kuşku Çabuk F. (2016). Mechanoreceptors of the ligaments and tendons around the knee. Clin. Anat. (New York, N.Y.) 29 (6), 789–795. 10.1002/ca.22743 27376635

[B5] Cavalcante M. L. Rodrigues C. J. Mattar R. Jr (2004). Mechanoreceptors and nerve endings of the triangular fibrocartilage in the human wrist. J. hand Surg. 29 (3), 432–435. 10.1016/j.jhsa.2004.01.001 15140485

[B6] Clanton T. O. Campbell K. J. Wilson K. J. Michalski M. P. Goldsmith M. T. Wijdicks C. A. (2014). Qualitative and quantitative anatomic investigation of the lateral ankle ligaments for surgical reconstruction procedures. J. bone Jt. Surg. Am. volume 96 (12), e98. 10.2106/JBJS.M.00798 24951749

[B7] Coetzee J. C. Ellington J. K. Ronan J. A. Stone R. M. (2018). Functional results of open Broström ankle ligament repair augmented with a suture tape. Foot ankle Int. 39 (3), 304–310. 10.1177/1071100717742363 29420055

[B8] Dalmau-Pastor M. Malagelada F. Kerkhoffs G. M. M. J. Manzanares M. C. Vega J. (2018). X-shaped inferior extensor retinaculum and its doubtful use in the Bröstrom-Gould procedure. Knee Surg. sports traumatology, Arthrosc. official J. ESSKA 26 (7), 2171–2176. 10.1007/s00167-017-4647-y 28710509

[B9] Feng S. M. Maffulli N. Oliva F. Wang A. G. Sun Q. Q. (2020). Arthroscopic remnant-preserving anterior talofibular ligament reconstruction does not improve mid-term function in chronic ankle instability. Injury 51 (8), 1899–1904. 10.1016/j.injury.2020.05.011 32536527

[B10] Fisher A. Bond A. Philpott M. D. G. Jayatilaka M. L. T. Lambert L. A. Fisher L. (2021). The anatomy of the anterior inferior tibiofibular ligament and its relationship with the Wagstaffe fracture. Foot ankle Surg. official J. Eur. Soc. Foot Ankle Surg. 27 (3), 291–295. 10.1016/j.fas.2021.01.003 33446454

[B11] Freeman M. A. Wyke B. (1966). Articular contributions to limb muscle reflexes. The effects of partial neurectomy of the knee-joint on postural reflexes. Br. J. Surg. 53 (1), 61–69. 10.1002/bjs.1800530116 5901916

[B12] Guelfi M. Zamperetti M. Pantalone A. Usuelli F. G. Salini V. Oliva X. M. (2018). Open and arthroscopic lateral ligament repair for treatment of chronic ankle instability: a systematic review. Foot ankle Surg. official J. Eur. Soc. Foot Ankle Surg. 24 (1), 11–18. 10.1016/j.fas.2016.05.315 29413768

[B13] Guillo S. Bauer T. Lee J. W. Takao M. Kong S. W. Stone J. W. (2013). Consensus in chronic ankle instability: aetiology, assessment, surgical indications and place for arthroscopy. Orthop. traumatology, Surg. Res. OTSR 99 (8 Suppl. l), S411–S419. 10.1016/j.otsr.2013.10.009 24268842

[B14] Han J. Qian S. Lian J. Wu H. Zheng B. Wu X. (2023). Modified classifications and surgical decision-making process for chronic anterior talofibular ligament injuries based on the correlation of imaging studies and arthroscopic findings. Int. Orthop. 47 (11), 2683–2692. 10.1007/s00264-023-05896-6 37477681

[B15] Katonis P. G. Assimakopoulos A. P. Agapitos M. V. Exarchou E. I. (1991). Mechanoreceptors in the posterior cruciate ligament. Histologic study on cadaver knees. Acta Orthop. Scand. 62 (3), 276–278. 10.3109/17453679108993609 2042472

[B16] Kobayashi T. Suzuki D. Kondo Y. Tokita R. Katayose M. Matsumura H. (2020). Morphological characteristics of the lateral ankle ligament complex. Surg. radiologic Anat. SRA 42 (10), 1153–1159. 10.1007/s00276-020-02461-3 32227271

[B17] Larkins C. G. Brady A. W. Aman Z. S. Dornan G. J. Haytmanek C. T. Clanton T. O. (2021). Evaluation of the intact anterior talofibular and calcaneofibular ligaments, injuries, and repairs with and without augmentation: a biomechanical robotic study. Am. J. sports Med. 49 (9), 2432–2438. 10.1177/03635465211018645 34110933

[B18] Pacheco J. Guerra-Pinto F. Araújo L. Flora M. Alçada R. Rocha T. (2021). Chronic ankle instability has no correlation with the number of ruptured ligaments in severe anterolateral sprain: a systematic review and meta-analysis. Knee Surg. sports traumatology, Arthrosc. official J. ESSKA 29 (11), 3512–3524. 10.1007/s00167-021-06610-y 33993320

[B19] Petrera M. Dwyer T. Theodoropoulos J. S. Ogilvie-Harris D. J. (2014). Short-to medium-term outcomes after a modified Broström repair for lateral ankle instability with immediate postoperative weightbearing. Am. J. sports Med. 42 (7), 1542–1548. 10.1177/0363546514530668 24769409

[B20] Rasmussen O. Tovborg-Jensen I. Boe S. (1982). Distal tibiofibular ligaments. Analysis of function. Acta Orthop. Scand. 53 (4), 681–686. 10.3109/17453678208992276 7102288

[B21] Stake I. K. Bryniarski A. R. Brady A. W. Miles J. W. Dornan G. J. Madsen J. E. (2023). Effect of posterior malleolar fixation on syndesmotic stability. Am. J. sports Med. 51 (4), 997–1006. 10.1177/03635465231151448 36779585

[B22] Tian J. Mok T. N. Sin T. H. Zha Z. Zheng X. Teng Q. (2022). Clinical outcomes of anterior tibiofibular ligament's distal fascicle transfer versus ligament reconstruction with InternalBrace™ for chronic ankle instability patients. Archives Orthop. trauma Surg. 142 (10), 2829–2837. 10.1007/s00402-021-04214-2 PMC9474461 34846587

[B23] Tourné Y. Mabit C. (2017). Lateral ligament reconstruction procedures for the ankle. Orthop. traumatology, Surg. Res. OTSR 103 (1S), S171–S181. 10.1016/j.otsr.2016.06.026 27871968

[B24] Vega J. Poggio D. Heyrani N. Malagelada F. Guelfi M. Sarcon A. (2020). Arthroscopic all-inside ATiFL's distal fascicle transfer for ATFL's superior fascicle reconstruction or biological augmentation of lateral ligament repair. Knee Surg. sports traumatology, Arthrosc. official J. ESSKA 28 (1), 70–78. 10.1007/s00167-019-05460-z 30888451

[B25] Waldrop N. E. 3rd Wijdicks C. A. Jansson K. S. LaPrade R. F. Clanton T. O. (2012). Anatomic suture anchor versus the Broström technique for anterior talofibular ligament repair: a biomechanical comparison. Am. J. sports Med. 40 (11), 2590–2596. 10.1177/0363546512458420 22962291

[B26] Woo B. J. Lai M. C. Koo K. (2020). Arthroscopic versus open broström-gould repair for chronic ankle instability. Foot ankle Int. 41 (6), 647–653. 10.1177/1071100720914860 32207336

[B27] Xiao L. Zheng B. Zhou Y. Hu D. Li J. Zheng X. (2022). Biomechanical study of arthroscopic all-inside anterior talofibular ligament suture augmentation repair, plus suture augmentation repair and anterior tibiofibular ligament's distal fascicle transfer augmentation repair. J. Clin. Med. 11 (17), 5235. 10.3390/jcm11175235 36079163 PMC9456712

[B28] Yang Y. Han J. Wu H. Zhi X. Lian J. Xu F. (2022). Arthro-Broström with endoscopic retinaculum augmentation using all-inside lasso-loop stitch techniques. BMC Musculoskelet. Disord. 23 (1), 795. 10.1186/s12891-022-05709-8 35987668 PMC9392268

[B29] Yeo E. D. Rhyu I. J. Kim H. J. Kim D. S. Ahn J. H. Lee Y. K. (2016). Can Bassett's ligament be removed? Knee Surg. sports traumatology, Arthrosc. official J. ESSKA 24 (4), 1236–1242. 10.1007/s00167-015-3903-2 26685686

[B30] Zimny M. L. St Onge M. Schutte M. (1985). Notes on technic. Stain Technol. 60 (5), 305–306. 10.3109/10520298509113929 2412319

